# A refined and updated health impact assessment of the Global Programme to Eliminate Lymphatic Filariasis (2000–2020)

**DOI:** 10.1186/s13071-022-05268-w

**Published:** 2022-05-28

**Authors:** Hugo C. Turner, Eric A. Ottesen, Mark H. Bradley

**Affiliations:** 1 MRC Centre for Global Infectious Disease Analysis, School of Public Health, Imperial College London, London, UK; 2 The Taskforce for Global Health, Decatur, Georgia; 3 Global Health Programs GlaxoSmithKline, London, UK

**Keywords:** Lymphatic filariasis, DALYs averted, Health impact, GPELF, Programme evaluation

## Abstract

**Background:**

Lymphatic filariasis (LF) is a neglected tropical disease (NTD). In 2000 the World Health Organization (WHO) established the Global Programme to Eliminate Lymphatic Filariasis (GPELF). A key component of this programme is mass drug administration (MDA). Between 2000 and 2020, the GPELF has delivered over 8.6 billion treatments to at-risk populations. The last impact assessment of the programme evaluated the treatments provided between 2000–2014. The goal of this analysis is to provide an updated health impact assessment of the programme, based on the numbers treated between 2000–2020.

**Methods:**

We updated and refined a previously established model that estimates the number of clinical manifestations and disability-adjusted life years (DALYs) averted by the treatments provided by the GPELF. The model comprises three different population cohorts that can benefit from MDA provided (those protected from acquiring infection, those with subclinical morbidity prevented from progressing and those with clinical disease alleviated). The treatment numbers were updated for all participating countries using data from the WHO. In addition, data relating to the estimated number of individuals initially at risk of LF infection were updated where possible. Finally, the DALY calculations were refined to use updated disability weights.

**Results:**

Using the updated model and corresponding treatment data, we projected that the total benefit cohort of the GPELF (2000–2020) would consist of approximately 58.5 million individuals and the programme would avert 44.3 million chronic LF cases. Over the lifetime of the benefit cohorts, this corresponded to 244 million DALYs being averted.

**Conclusion:**

This study indicates that substantial health benefits have resulted from the first 20 years of the GPELF. It is important to note that the GPELF would have both additional benefits not quantified by the DALY burden metric as well as benefits on other co-endemic diseases (such as soil-transmitted helminths, onchocerciasis and scabies)—making the total health benefit underestimated. As with the past impact assessments, these results further justify the value and importance of continued investment in the GPELF.

**Graphical Abstract:**

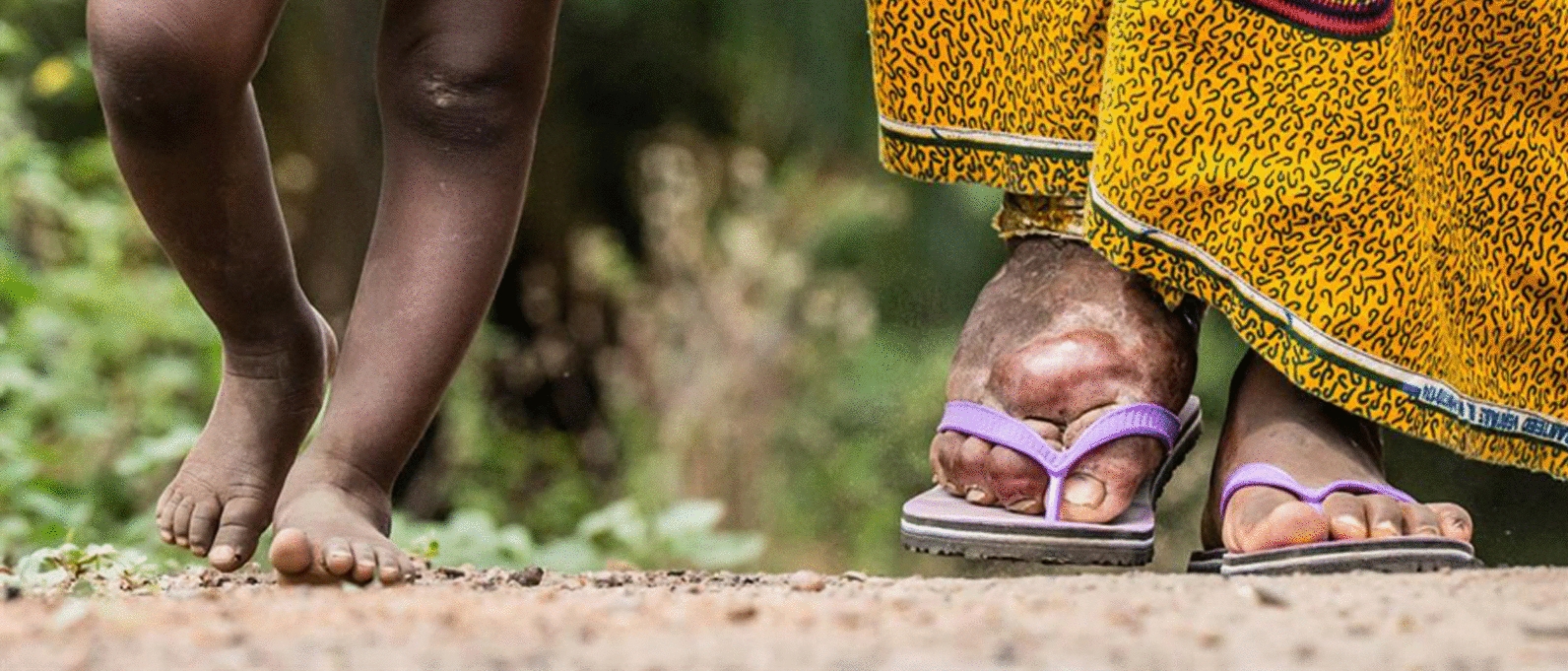

## Background

Lymphatic filariasis (LF), also known as elephantiasis, is a neglected tropical disease (NTD). In 1997, the World Health Assembly passed Resolution 50.29, calling for the elimination of LF as a public health problem [1]. Following on from this, in 2000 the World Health Organization (WHO) established the Global Programme to Eliminate Lymphatic Filariasis (GPELF) with the aspiration of eliminating the disease as a public health problem by 2020 [2, 3]. To date, the GPELF has been implemented by 69 out of 73 member states [4]. For most of the period 2000–2020, LF has been treated with mass drug administration (MDA) using a combination of either albendazole and ivermectin (in areas co-endemic with onchocerciasis) or albendazole and diethylcarbamazine (DEC) elsewhere. Following an extensive evaluation of the efficacy, safety and acceptability of a three-drug treatment regimen, in 2017 the WHO introduced IDA (ivermectin, DEC and albendazole) as an additional strategy to accelerate progress to elimination [5]. This MDA strategy has been shown to be feasible at a large scale and cost-effective [6–8]. A major factor in the success of the programmes is the fact that the drugs used are largely donated by pharmaceutical partners [9].

We have conducted two previous health and economic assessments of the GPELF, the first for the period 2000–2007 and the second for 2000–2014 [10, 11]. The most recent analysis of the GPELF projected that due to the treatments given between 2000–2014, 36 million clinical cases and 175 million disability-adjusted life years (DALYs) will potentially be averted [11]. The goal of this analysis is to provide an update of this health impact assessment based on the numbers treated within the programme between 2000–2020 and uses updated disability weights for LF morbidity.

## Methods

A detailed summary of the impact model is provided in Turner et al. [11]. To briefly summarize, the model comprises three different benefit cohorts that can benefit from the MDA provided:

- Benefit cohort 1: Individuals protected from acquiring infection and, therefore, subsequently protected from any clinical disease: The reductions in the number at risk were approximated for each country using a model defining reductions in risk of infection among cohorts of treated populations following each treatment round [12]. The model ‘zeroed out’ the at-risk population in the country once it had passed the transmission assessment survey [12].
- Benefit cohort 2: Individuals with existing subclinical morbidity who were protected from progression to clinical disease by the MDAs.
- Benefit cohort 3: Individuals with existing clinical disease for whom some morbidity was alleviated as a result of the MDAs.

The benefit cohorts quantify the individuals who are projected to benefit directly in terms of prevented or alleviated clinical disease by the treatments given between 2000–2020, i.e. they capture the long-term health benefits of these treatments beyond the year 2020, over the lifetime of the benefit cohorts. The different benefit cohorts are mutually exclusive, and individuals do not move between them. Within the model, the number of uniquely treated individuals in any one country was assumed to be the maximum number of individuals treated in any single MDA for each country.

The following key changes were made from the previous analysis.

- Using data from the WHO PCT databank [13], treatment numbers were updated for all participating countries including any additional countries that entered the GPELF during the period 2015–2020. Based on this it was calculated that between 2000–2020, 8.626 billion treatments were provided (compared to 5.626 billion treatments between 2000–2014) (Table 1).
- Data relating to the number at risk of infection within a country were updated where necessary—including for countries not included within the previous analysis or counties where updated information was available [13]. We used the data that corresponded to the year when mapping was completed for a given country. Note that in some cases the estimated number of individuals initially at risk of infection has decreased for a country compared to the 2000–2014 analysis because of updated mapping activities (Table 1).
- The DALY calculations were refined to use updated disability weights—including employing a disability weight for acute adenolymphangitis (ADL) episodes (inflammation of the lymph vessels or glands often accompanied by pain, fever and swelling [14]) (Table 2). Since chronic disease and ADL episodes coexist, we have accounted for the overlap in our estimation of the burden by using the multiplicative adjustment method [15]. The reason that the DALYs related to ADL episodes were previously ignored was that ADL episodes did not have a corresponding disability weight at the time of the previous studies. Note that other relevant disability weights changed because of updates and additional survey data within the Global Burden of Disease (GBD) studies (particularly between the 2010 and 2013 studies).

**Table 1:** Summary of the number of treatments provided and the population at risk of infection

	Population at risk of infection when national programmes began (millions)		Total number of treatments provided within the analysis (millions)	
	2000–2014 analysis	2000–2020 analysis	2000–2014 analysis	2000–2020 analysis
AFR	424.91	490.68	876.37	2024.70
AMR	14.10	14.14	46.95	65.98
EMR	22.81	12.59	15.96	24.46
SEAR	901.87	902.13	4494.47	6284.80
WPR	45.22	45.08	192.59	225.97
Total	1408.92	1464.62	5626.33	8625.91

The most recent estimates are taken from the PCT databank [12]

See Turner et al. [11] for details of the 2000–2014 analysis

*AMR *Region of the Americas, *AFR *African Region*, EMR *Eastern Mediterranean Region*, WPR *Western Pacific Region*, SEAR *Southeast Asia Region

**Table 2:** Summary of the sensitivity analysis

Parameter		Baseline hydrocele average estimate (range)	Baseline lymphedema average estimate (range)	Sources
Pre-control burden				
Percentage of the at-risk population that develop clinical disease		2.08% (1.04%)	1.25% (0.63%)	[16]
Disability weights				
Disability weights related to chronic disease		0.128 (0.086–0.180)	0.109 (0.073–0.154)	[17]
Disability weight for ADL episodes		0.051 (0.032–0.074)	0.051 (0.032–0.074)	[17]
Disease progression & incidence rates				
Percentage of clinical patients who experience ADL episodes per year		70% (45–90%)	95% (90–95%)	[26–34]
Frequency of ADL episodes for clinical patients (in absence of MDA)		2 (0–7) per year	4 (0–7) per year	[26–34]
Average duration of an ADL episode		4 (1–9) days	4 (1–9) days	[26–34]
Mean age of the benefit cohorts (years)		Cohort 1: 20 (30) Cohort 2: 20 (30) Cohort 3: 30 (40)	Cohort 1: 20 (30) Cohort 2: 20 (30) Cohort 3: 30 (40)	
Impact of treatment				
The reduction in transmission experienced by the treated population (Benefit cohort 1)		Year 1: 50% (35%) Year 2: 75% (53%) Year 3: 88% (62%) Year 4: 94% (66%) Year 5 95% (67%)	Year 1: 50% (35%) Year 2: 75% (53%) Year 3: 88% (62%) Year 4: 94% (66%) Year 5 95% (67%)	[12]
Reduction in the frequency of ADL episodes by MDA (Benefit cohort 3)		50% (15–88%)	50% (15–88%)	[35–37]
Percentage of chronic disease alleviated by MDA (Benefit cohort 3)		10% (0–20%)	15% (0–30%)	[35, 38–43]

Based on Chu et al. [10], though updated where appropriate

*ADL *acute adenolymphangitis, *DALY *disability-adjusted life year, * MDA *mass drug administration

The counterfactual scenario was no treatments being provided to these populations. The time horizon was the lifetime of individuals in the benefit cohorts [11].

Table 1 summarises the differences in the number of treatments provided and the population at risk of infection between this and the 2000–2014 analysis.

### Sensitivity analysis

Sensitivity analysis was performed on the following areas: (i) the pre-control burden of LF, (ii) the DALY disability weights, (iii) the disease progression/incidence rates and (iv) the impact of treatment (Table 2). The parameters and ranges investigated are shown in Table 2.

## Results

Using the model and corresponding treatment data, we projected that the total benefit cohort of the GPELF (2000–2020) would consist of approximately 58.5 million individuals, i.e. these individuals would have experienced the health benefits related to LF due to the MDA provided by the GPELF (Table 3). Of these, 26 million (44%) were in Benefit cohort 1 (those who would have acquired LF and subsequently progressed to clinical disease but were protected from infection due to reductions in transmission by MDA). The remaining 32.5 million were individuals who were already infected at the time of MDA treatment but benefited from halted disease progression [Benefit cohort 2: 14.8 million (25%)] or those with clinical disease who experienced some alleviated morbidity [Benefit cohort 3: 17.7 million (30%)] (Table 3).

**Table 3:** Total population size of the benefit cohorts and numbers of cases of chronic disease averted

	Benefit cohort 1: Protected from acquiring infection and, therefore, subsequently protected from any clinical disease (millions)	Benefit cohort 2: Subclinical morbidity prevented from progressing (millions)	Benefit cohort 3: Clinical disease improved (millions)	Total (millions)
Population size				
Hydrocele	16.23	9.26	9.37	34.86
Lymphedema	9.74	5.56	8.30	23.60
Total	25.97	14.82	17.67	58.46
Cases of chronic disease averted				
Hydrocele	16.23	9.26	1.82	27.31
Lymphedema	9.74	5.56	1.64	16.93
Total	25.97	14.82	3.46	44.25

Note that the benefit cohorts quantify the individuals who are projected to benefit directly in terms of prevented or alleviated clinical disease by the treatments given between 2000–2020, i.e. they capture the long-term health benefits of these treatments beyond the year 2020

### Health benefits

Based on the three benefit cohorts we estimated that the treatments provided between 2000–2020 would prevent 44.3 million chronic cases of either hydrocele or lymphedema (Table 3). When projecting the health benefits over the lifetimes of the benefit cohorts, this corresponded to averting 1997 million years of life lived with chronic disease and 5858 million ADL episodes (Table 4). With the baseline parameters, this projected lifetime health benefit translates into averting approximately 244 million DALYs (Table 4).

**Table 4:** Health impact over the lifetime of the benefit cohorts

	Person-years of chronic LF prevented (millions)	ADL cases prevented (millions)	DALYs averted (millions)
AFR	595.23	1746.72	72.74
AMR	19.82	58.25	2.42
EMR	14.55	43.15	1.78
SEAR	1309.32	3841.49	160.00
WPR	57.58	168.86	7.04
Total	1996.50	5858.48	243.98

**Fig. 1:**
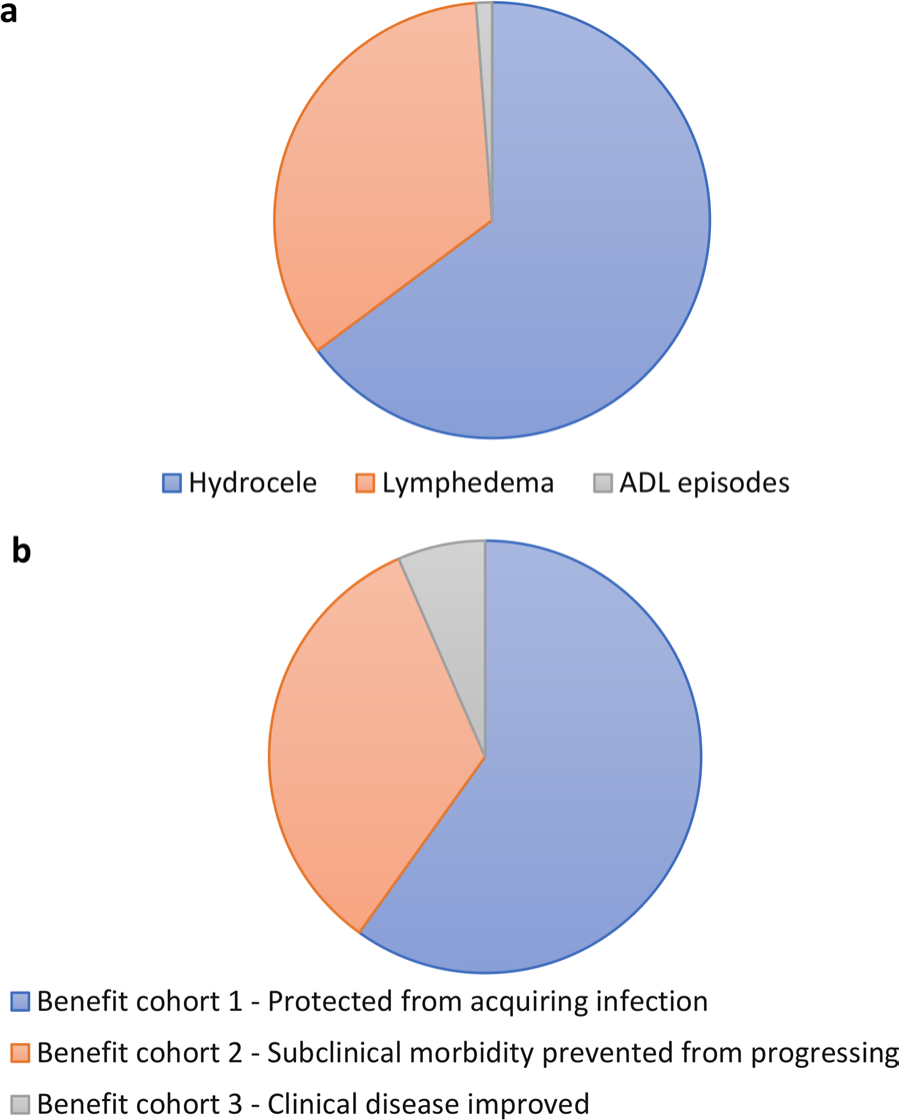
Breakdown of the total number of DALYs averted. **a** Stratified by morbidity manifestation. **b** Stratified by benefit cohort. *ADL* Acute adenolymphangitis. *DALY* Disability-adjusted life year

Prevented hydrocele contributed most to the projected total health impact (Fig. 1a). This is because the baseline proportion of clinical disease patients with hydrocele was assumed to be higher than in those with lymphedema (62.5% vs. 37.5% [16]) and the higher disability weight related to hydrocele (Table 2). Prevented ADL episodes contributed little to the number of DALYs averted (1%). This is because the DALY weight is only applied to the very small proportion of the year they are experiencing ADL episodes, so overall it is small compared to chronic morbidity.

As with the previous analysis, the total health impact was the smallest within the alleviated clinical disease cohort (Benefit cohort 3) despite its comparable population size (Fig. 1 and Tables 3 and 4). This is due to the fact that the majority of this cohort only experience reductions in the frequency of acute ADL episodes and not alleviated chronic disease (due to the assumed effects of the drugs on morbidity—Table 2). The largest contribution to the health impact comes from Benefit cohort 1 (individuals who would have acquired LF and subsequently would progress to clinical disease but were protected from infection because of the reductions in transmission by MDA) (Fig. 1b).

The majority of the health impact was due to the considerable scope of the MDA programmes in the Southeast Asia region (largely due to India) (Table 4). A very notable health impact also resulted from the treatments in the Africa Region.

### Sensitivity analysis

To explore how sensitive the projections were to variation in the input parameters and assumptions, the model projections were subjected to univariate sensitivity analysis (see Table 2).

**Fig. 2:**
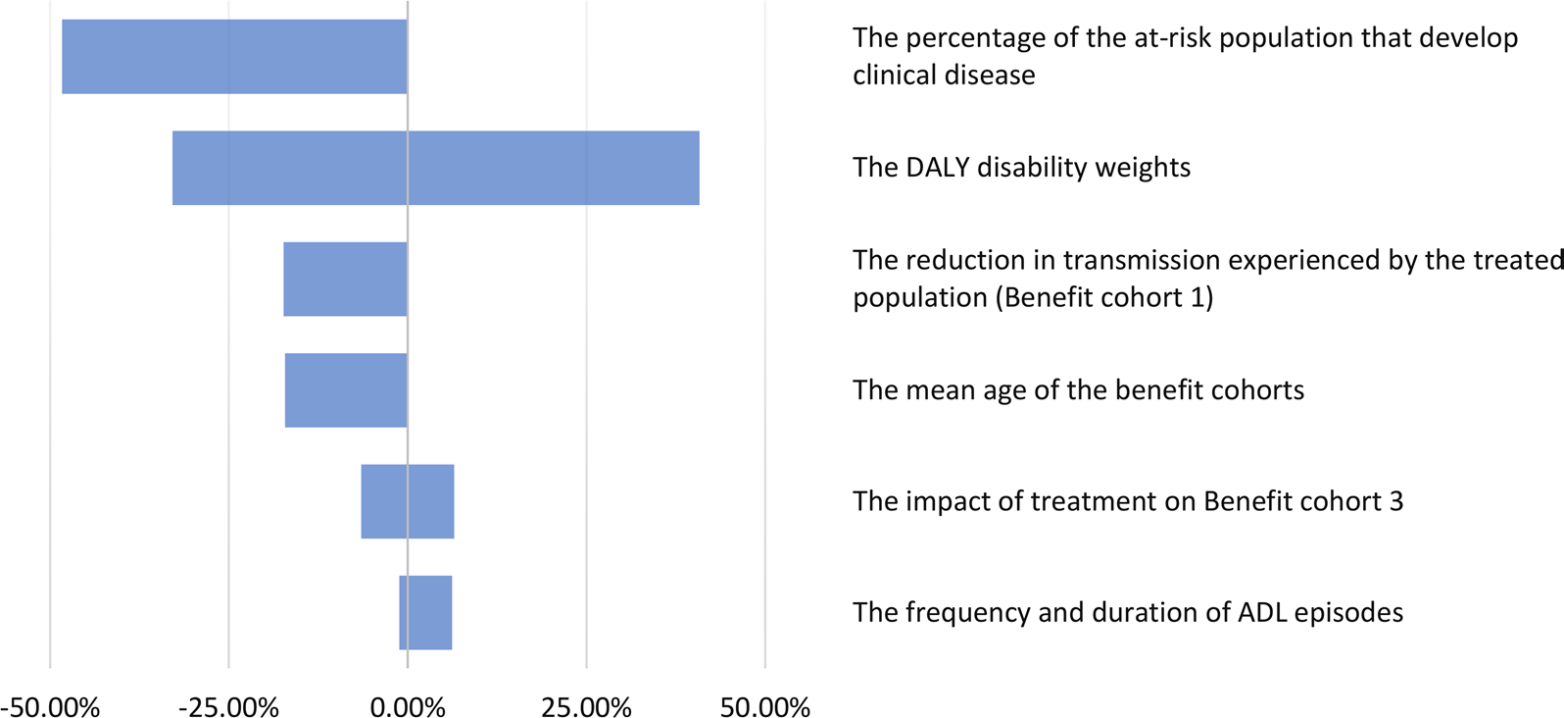
Tornado plot illustrating the impact of the sensitivity analysis on the estimated total health impact (number of DALYs averted) of the GPELF (2000–2020). The parameter ranges are presented in Table 2

Overall, the projected impact on the overall health benefits was found to be robust (Fig. 2). As expected, the projected benefits were related to the assumed percentage of the at-risk population that develops clinical disease. The number of DALYs averted was reduced by 17% when assuming more conservative reductions in transmission and 17% when increasing the mean age of the benefit cohorts by 10 years. When assuming that MDA has no impact regarding alleviating established disease (i.e. no benefit for cohort 3), the number of DALYs averted only decreased by 6%. The results were not sensitive to the assumptions regarding the incidence and duration of ADL episodes. The number of DALYs averted was also sensitive to the assumed disability weight [17]. However, even when using the lower bound for the disability weights (Table 2), we still projected that 164 million DALYs would be averted.

## Discussion

The updated and refined model projects that due to the MDA provided by the GPELF between 2000–2020, 44.3 million chronic cases would be averted (Table 3). Over the lifetime of the benefit cohorts, this corresponded to 244 million DALYs being averted (Table 4). This updated analysis further supports the implication that administering MDA to such a large population has produced substantial health benefits over the first 20 years of the programme. Although the estimates were sensitive to certain parameters, the notable health impact appeared robust within the sensitivity analysis.

These impact estimates are important as donors and member states need evidence that there is a positive impact of the GPELF to justify the continued investment to the programme from their limited budgets. They can also provide an important input to cost-effectiveness analysis—investigating the value for money (cost per DALY averted) of the programme [7].

It is also important to note that the GPELF uses albendazole and ivermectin, which are highly effective, broad-spectrum anti-parasitic drugs. Consequently, the programme will have notable ancillary benefits on other parasitic diseases that are common in the populations targeted by the GPELF (described in more detail in Box 1 and elsewhere [18]).

Within the analysis, we estimated that 44.3 million chronic cases of LF would be averted. In comparison, de Vlas et al. [19] estimated 46.4 million new cases of irreversible disease would be prevented between 2011–2030 if the 2020 goals were achieved for LF. One reason for this difference is that the targets set within the 2020 goals have not been achieved in every country. In addition, within this analysis we are quantifying the health benefit over the lifetime of the benefit cohort and not only up to 2030.

Although the estimates presented in this study support the notable long-term health impact that MDA will have, it is also important to note that millions of chronic cases of LF will remain, even after MDA has successfully interrupted transmission. This highlights that to truly eliminate LF as a public health problem there is also a need for morbidity management strategies as well as MDA within the GPELF.

### Limitations and key sources of uncertainty

The limitations of the model and analysis are highlighted in Turner et al. [11].

One of the largest limitations of this study is the uncertainty regarding the pre-control prevalence of LF and the baseline burden of clinical disease. The assumptions within this analysis were that on average 10% of the initial at-risk population will be infected, of which one-third bear chronic infection (62.5% with hydrocoele and 37.5% with lymphoedema). These were based on a review by Michael et al. [16]. However, a limitation of this study is that the analysis applies these estimates uniformly to the at-risk populations, ignoring the varying distribution of disease and heterogeneity in transmission. An analysis using the same model structure and parameters evaluating the pre-control health burden of LF estimated that approximately 129 million were infected with LF, of whom 43 million had clinical disease [20]. This corresponded to a DALY burden of 5.25 million [20]. However, by comparison, the current GBD study estimates that in 2000 there were 261 million infected with LF with a corresponding burden of 4.99 million DALYs [21]. The similar DALY burden implies that the GBD study estimates are assuming that there is a higher prevalence of infection, but that a lower proportion develops clinical disease than assumed in our study, which in this case balances out to produce a similar clinical burden estimate. A lower pre-control burden was considered in our sensitivity analysis (Table 2). Although this obviously decreased the impact estimates, the health gains were still appreciable.

Furthermore, the limited number of regional/country-specific primary data available somewhat limited the breadth of this analysis. Due to this lack of data, many of the LF disease-specific parameters (such as the baseline prevalence) were attributed a global standardized estimate.

As with the previous version of the analysis, it is not possible to estimate the number of uniquely treated individuals across multiple MDA rounds. Consequently, to be conservative, the number of uniquely treated individuals in any one country was assumed to be the maximum number of individuals treated in any single MDA for each country—which in most cases will likely underestimate the number of uniquely treated individuals. A further limitation of this analysis was that population growth was not considered.

Within the model the different drug regimens used within the GPELF were assumed to be equally effective in their impact on both LF disease and filarial infections themselves [22]. However, it should be noted that in a few central African countries where the Loa Loa prevalence precludes the use of ivermectin for safety reasons, twice-annual albendazole monotherapy is used for LF elimination. In addition, in some countries, DEC monotherapy was used for certain years [13]. The potential lower impact of monotherapy was not accounted for in this analysis. In addition, although treatments with the IDA regimen were included in the analysis, the model used to calculate the health benefit resulting from reduced transmission (Benefit cohort 1) is not parametrised for IDA. These treatments were therefore conservatively assumed to have the same impact as a standard dual drug treatment. That said, globally between 2000–2020, there have only been approximately 60.6 million treatments with the IDA regimen – compared to the overall > 8.6 billion treatments included in the analysis [12]. Consequently, although the IDA regimen is an important new intervention in terms of achieving LF elimination, its past use to date would have little impact on this 2000–2020 health impact assessment of the GPELF.

Finally, it is important to consider that the DALY metric may not fully capture the health benefits of the GPELF. For example, the universal disability weights used for DALY calculations do not account for how the local context could influence the burden of a disease, and in this context, how the burden of LF-related morbidity could be worse for those who are living in poverty [20, 23]. The DALY disability weights also have limitations regarding not fully accounting for the psycho-social impact of illness for certain disease sequelae and are a narrower measure of health gains compared to quality-of-life measures. Ton et al. [24] highlighted that the DALY burden attributable to LF could increase significantly if the depression experienced by LF patients was also quantified. In addition, DALYs often fail to account for the burden experienced by patients’ caregivers [20, 24, 25], and it was assumed that clinical LF morbidity was not associated with any excess mortality—both of which could underestimate the burden of LF and the consequent benefit of the GPELF. Therefore, further studies would be of value to quantify the broader socio-economic impact of preventing LF morbidity by capturing the impact on the patients’ and their caregivers’ quality of life [25].

## Conclusions

Despite the limitations of any such global health analysis, this study indicates that substantial health benefits have resulted from the first 20 years of the GPELF. We projected that due to the treatments provided between 2000–2020, 44.3 million chronic cases and 244 million DALYs would be averted over the lifetime of the benefit cohorts. The results were subjected to sensitivity analysis and were most sensitive to the assumed pre-control burden of clinical morbidity and the disability weights used. It is important to note that the GPELF also has had both additional benefits not quantified by the DALY burden metric as well as benefits from its effects on other co-endemic diseases (such as soil-transmitted helminths, onchocerciasis and scabies)—making the total health benefit even greater than that presented here. As with the past impact assessments, these results further justify both the value and the importance of continued investment in the GPELF.

Box 1 Ancillary benefits (adapted from [11])

**Table:** 

**Benefit for people with intestinal parasites:** Albendazole is also used for the control of soil-transmitted helminths (STH) and ivermectin has been shown to have anti-parasitic action against several intestinal parasites of concern. Consequently, the GPELF is expected to have an impact on the prevalence and intensity of common STH infections. It is important to note that LF programmes co-administrating albendazole and ivermectin will have a much higher impact on trichuris [44] than dedicated STH control programmes using single-drug treatments of albendazole or mebendazole [45]. Furthermore, the GPELF community-wide treatment programmes will have a higher impact on hookworm since the majority of worms are harboured by adults [45–47]. This community-wide approach also serves to address at least some of the needs for women of childbearing age living in hookworm endemic areas where anaemia is a concern during pregnancy [48, 49].	
**Benefit for people with scabies:** Ivermectin is an effective treatment for scabies and can cause the community prevalence to fall dramatically after a few rounds of treatment [50]. Cured individuals show improvements in sleep patterns and overall wellbeing and decreased incidence of skin infections and renal disease [51].	
**Benefit for co-endemic onchocerciasis areas:** Because of its broad geographic range, the GPELF has brought ivermectin treatment to millions of people living in onchocerciasis-endemic areas not previously targeted by onchocerciasis control programmes (as these programmes previously focused primarily on communities where the prevalence of onchocerciasis exceeds 40%) [52]. The GPELF is therefore likely contributing significantly to the elimination of onchocerciasis transmission.

## Data Availability

Data are available on reasonable request.

## Abbreviations

ADL: Acute adenolymphangitis
DALYs: Disability-adjusted life years
DEC: Diethylcarbamazine
GBD: Global burden of disease
GPELF: Global Programme to Eliminate Lymphatic Filariasis
LF: Lymphatic filariasis
MDA: Mass drug administration
NTD: Neglected tropical disease
STH: Soil-transmitted helminths
WHO: World Health Organization

## References

[1] WHA50.29: Elimination of lymphatic filariasis as a public health problem. http://www.who.int/neglected_diseases/mediacentre/WHA_50.29_Eng.pdf. Accessed 25 March 2022

[2] World Health Organization: Global Programme to Eliminate Lymphatic Filariasis. http://www.who.int/lymphatic_filariasis/elimination-programme/en/. Accessed 25 March 2022

[3] World Health Organization. Global Programme to Eliminate Lymphatic Filariasis: progress report 2000–2009 and strategic plan 2010–2020. World Health Organization. 2010; http://apps.who.int/iris/handle/10665/44473 Accessed 25 March 2022

[4] World Health Organization (2021). Global programme to eliminate lymphatic filariasis: progress report, 2020. Wkly Epidemiol Rec.

[5] World Health Organization: Guideline: alternative mass drug administration regimens to eliminate lymphatic filariasis. World Health Organization. 2017. https://www.ncbi.nlm.nih.gov/books/NBK487830/ Accessed 25 March 2022 29565523

[6] Gedge LM, Bettis AA, Bradley MH, Hollingsworth TD, Turner HC (2018). Economic evaluations of lymphatic filariasis interventions: a systematic review and research needs. Parasit Vectors.

[7] Turner HC, Bettis AA, Chu BK, McFarland DA, Hooper PJ, Mante SD (2017). Investment success in public health: An analysis of the cost-effectiveness and cost-benefit of the Global Programme to Eliminate Lymphatic Filariasis. Clin Infect Dis.

[8] Turner HC, Stolk WA, Solomon AW, King JD, Montresor A, Molyneux DH (2021). Are current preventive chemotherapy strategies for controlling and eliminating neglected tropical diseases cost-effective?. BMJ Global Health.

[9] World Health Organization: Contribution of pharmaceutical companies to the control of neglected tropical diseases. http://www.who.int/neglected_diseases/pharma_contribution/en/. Accessed 25 March 2022

[10] Chu BK, Hooper PJ, Bradley MH, McFarland DA, Ottesen EA (2010). The economic benefits resulting from the first 8 years of the Global Programme to Eliminate Lymphatic Filariasis (2000–2007). PLoS Negl Trop Dis.

[11] Turner HC, Bettis AA, Chu BK, McFarland D, Hooper PJ, Ottesen EA (2016). The health and economic benefits of the global programme to eliminate lymphatic filariasis (2000–2014). Infect Dis Poverty.

[12] Hooper PJ, Chu BK, Mikhailov A, Ottesen EA, Bradley M (2014). Assessing progress in reducing the at-risk population after 13 years of the global programme to eliminate lymphatic filariasis. PLoS Negl Trop Dis.

[13] World Health Organization: PCT databank: Lymphatic filariasis. http://www.who.int/neglected_diseases/preventive_chemotherapy/lf/en/. Accessed 25 March 2022

[14] World Health Organization. Lymphatic filariasis: managing morbidity and preventing disability: an aide-mémoire for national programme managers. World Health Organization. 2021. https://www.who.int/publications/i/item/lymphatic-filariasis-managing-morbidity-and-preventing-disability-an-aide-m%C3%A9moire-for-national-programme-managers-2nd-ed. Accessed 25 March 2022

[15] van Baal PHM, Hoeymans N, Hoogenveen RT, de Wit GA, Westert GP (2006). Disability weights for comorbidity and their influence on health-adjusted life expectancy. Popul Health Metrics.

[16] Michael E, Bundy DA, Grenfell BT (1996). Re-assessing the global prevalence and distribution of lymphatic filariasis. Parasitology.

[17] Global Burden of Disease Study 2019 (GBD 2019) Disability Weights. http://ghdx.healthdata.org/record/ihme-data/gbd-2019-disability-weights. Accessed 25 March 2022

[18] Ottesen EA, Hooper PJ, Bradley M, Biswas G (2008). The global programme to eliminate lymphatic filariasis: health impact after 8 years. PLoS Negl Trop Dis.

[19] de Vlas SJ, Stolk WA, le Rutte EA, Hontelez JAC, Bakker R, Blok DJ (2016). Concerted Efforts to Control or Eliminate Neglected Tropical Diseases: How Much Health Will Be Gained?. PLoS Negl Trop Dis.

[20] Mathew CG, Bettis AA, Chu BK, English M, Ottesen EA, Bradley MH (2019). The Health and Economic Burdens of Lymphatic Filariasis Prior to Mass Drug Administration Programs. Clin Infect Dis.

[21] Institute for Health Metrics and Evaluation: GBD Results Tool. http://ghdx.healthdata.org/gbd-results-tool. Accessed 25 March 2022

[22] Ramaiah KD, Ottesen EA (2014). Progress and Impact of 13 Years of the Global Programme to Eliminate Lymphatic Filariasis on Reducing the Burden of Filarial Disease. PLoS Negl Trop Dis.

[23] King CH, Bertino AM (2008). Asymmetries of poverty: why global burden of disease valuations underestimate the burden of neglected tropical diseases. PLoS Negl Trop Dis.

[24] Ton TG, Mackenzie C, Molyneux DH (2015). The burden of mental health in lymphatic filariasis. Infect Dis Poverty.

[25] Caprioli T, Martindale S, Mengiste A, Assefa D, H/Kiros F, Tamiru M (2020). Quantifying the socio-economic impact of leg lymphoedema on patient caregivers in a lymphatic filariasis and podoconiosis co-endemic district of Ethiopia. PLoS Negl Trop Dis.

[26] Krishnamoorthy K (1999). Estimated costs of acute adenolymphangitis to patients with chronic manifestations of bancroftian filariasis in India. Indian J Public Health.

[27] Ramaiah KD, Ramu K, Kumar KN, Guyatt H (1996). Epidemiology of acute filarial episodes caused by Wuchereria bancrofti infection in two rural villages in Tamil, Nadu, south India. Trans R Soc Trop Med Hyg.

[28] Pani SP, Yuvaraj J, Vanamail P, Dhanda V, Michael E, Grenfell BT (1995). Episodic adenolymphangitis and lymphoedema in patients with bancroftian filariasis. Trans R Soc Trop Med Hyg.

[29] Ramaiah KD, Radhamani MP, John KR, Evans DB, Guyatt H, Joseph A (2000). The impact of lymphatic filariasis on labour inputs in southern India: results of a multi-site study. Ann Trop Med Parasitol.

[30] Babu BV, Nayak AN, Dhal K (2005). Epidemiology of episodic adenolymphangitis: a longitudinal prospective surveillance among a rural community endemic for bancroftian filariasis in coastal Orissa. India BMC Public Health.

[31] Gyapong JO, Gyapong M, Adjei S (1996). The epidemiology of acute adenolymphangitis due to lymphatic filariasis in northern Ghana. Am J Trop Med Hyg.

[32] Kessel JF (1957). Disabling effects and control of filariasis. Am J Trop Med Hyg.

[33] Gasarasi DB, Premji ZG, Mujinja PG, Mpembeni R (2000). Acute adenolymphangitis due to bancroftian filariasis in Rufiji district, south east Tanzania. Acta Trop.

[34] Sabesan S, Krishnamoorthy K, Pani SP, Panicker KN (1992). Mandays lost dueto repeated attacks of lymphatic filariasis. Trends in Life Sciences.

[35] Ciferri F, Siliga N, Long G, Kessel JF (1969). A filariasis-control program in American Samoa. Am J Trop Med Hyg.

[36] Fan PC, Peng HW, Chen CC (1995). Follow-up investigations on clinical manifestations after filariasis eradication by diethylcarbamazine medicated common salt on Kinmen (Quemoy) Islands, Republic of China. J Trop Med Hyg.

[37] Das L, Subramanyam Reddy G, Pani S (2003). Some observations on the effect of Daflon (micronized purified flavonoid fraction of Rutaceae aurantiae) in bancroftian filarial lymphoedema. Filaria J.

[38] Bockarie MJ, Tisch DJ, Kastens W, Alexander ND, Dimber Z, Bockarie F (2002). Mass treatment to eliminate filariasis in Papua New Guinea. N Engl J Med.

[39] Partono F (1985). Treatment of elephantiasis in a community with timorian filariasis. Trans R Soc Trop Med Hyg.

[40] Partono F, Purnomo OS, Soewarta A (1981). The long term effects of repeated diethylcarbamazine administration with special reference to microfilaraemia and elephantiasis. Acta Trop.

[41] Meyrowitsch DW, Simonsen PE, Makunde WH (1996). Mass diethylcarbamazine chemotherapy for control of bancroftian filariasis through community participation: comparative efficacy of a low monthly dose and medicated salt. Trans R Soc Trop Med Hyg.

[42] Mackenzie CD, Lazarus WM, Mwakitalu ME, Mwingira U, Malecela MN (2009). Lymphatic filariasis: patients and the global elimination programme. Ann Trop Med Parasitol.

[43] March HN, Laigret J, Kessel JF, Bambridge B (1960). Reduction in the prevalence of clinical filariasis in Tahiti following adoption of a control program. Am J Trop Med Hyg.

[44] Knopp S, Mohammed KA, Speich B, Hattendorf J, Khamis IS, Khamis AN (2010). Albendazole and mebendazole administered alone or in combination with ivermectin against *Trichuris trichiura*: a randomized controlled trial. Clin Infect Dis.

[45] Truscott J, Turner H, Anderson R (2015). What impact will the achievement of the current World Health Organisation targets for anthelmintic treatment coverage in children have on the intensity of soil transmitted helminth infections?. Parasit Vectors.

[46] Turner HC, Truscott JE, Bettis AA, Shuford KV, Dunn JC, Hollingsworth TD (2015). An economic evaluation of expanding hookworm control strategies to target the whole community. Parasit Vectors.

[47] Pion SD, Chesnais CB, Bopda J, Louya F, Fischer PU, Majewski AC (2015). The impact of two semiannual treatments with albendazole alone on lymphatic filariasis and soil-transmitted helminth infections: a community-based study in the republic of congo. Am J Trop Med Hyg.

[48] Gyorkos TW, Montresor A, Belizario V, Biggs B-A, Bradley M, Brooker SJ (2018). The right to deworming: The case for girls and women of reproductive age. PLOS Neglected Tropical Diseases.

[49] World Health Organization: Reaching girls and women of reproductive age with deworming: report of the Advisory Group on deworming in girls and women of reproductive age, Rockefeller Foundation Bellagio Center, Bellagio, Italy 28–30 June 2017. World Health Organization. 2018. https://apps.who.int/iris/handle/10665/259962. Accessed 25 March 2022

[50] Heukelbach J, Winter B, Wilcke T, Muehlen M, Albrecht S, de Oliveira FA (2004). Selective mass treatment with ivermectin to control intestinal helminthiases and parasitic skin diseases in a severely affected population. Bull World Health Organ.

[51] Lawrence G, Leafasia J, Sheridan J, Hills S, Wate J, Wate C (2005). Control of scabies, skin sores and haematuria in children in the Solomon Islands: another role for ivermectin. Bull World Health Organ.

[52] Boatin BA, Richards FO (2006). Control of onchocerciasis. Adv Parasitol.

